# Epistaxis in Pregnant Women in the Covid-19 Era

**DOI:** 10.22038/ijorl.2024.76230.3553

**Published:** 2024

**Authors:** Mustafa Shamkhi Abbood, Eftekhar Shamkhee, Samah Abbas Hammadi

**Affiliations:** 1 *Department of General Surgery, College of Medicine, Ibn Sina University of Medical and Pharmaceutical Sciences, Baghdad, Iraq.*; 2 *Department of Gynecology, College of Medicine, Ibn Sina University of Medical and Pharmaceutical Sciences, Baghdad, Iraq.*; 3 *Department of Otolaryngology, College of Medicine, Al-Nahrain University, Baghdad, Iraq.*

**Keywords:** COVID-19, Epistaxis, Nasal bleeding, Pregnancy

## Abstract

**Introduction:**

Epistaxis is common throughout pregnancy and is usually not a cause for concern; severe nosebleeds are rare. An increased rate of nasal bleeding was observed during the COVID-19 epidemic.

**Materials and Methods:**

The study sample comprised 3,362 pregnant women who sought care at the Gynecologic and Obstetrics Department/ Al-Yarmouk Teaching Hospital and Al-Karkh Maternity Hospital between May 2020 and April 2021. All were asked to fill out a questionnaire.

**Results:**

Nine hundred forty-one pregnant women experienced an episode of epistaxis during the last pregnancy. One thousand seven hundred forty-eight pregnant women had a corona-positive history. Pregnant women with a positive history of coronavirus infection have a higher incidence of epistaxis (612 pregnant women) than pregnant women with a corona-negative history (329 pregnant women) P value (0.039%).

**Conclusions:**

Oxygen and blood-thinning drugs are the leading causes of the increased rate of nosebleeds among pregnant women during the Corona pandemic.

## Introduction

Epistaxis is common throughout pregnancy and is usually not a cause for concern. According to one study, up to 20% of women will develop a nosebleed when pregnant, while only about 6% get nosebleeds out of pregnancy (1). Increased blood volume and hormonal changes are the most common causes of epistaxis during pregnancy (1). Epistaxis exhibits a prevalence that is about thrice higher in pregnant women compared to their non-pregnant counterparts and is typically self-limiting (2).

Pregnant ladies are more likely to get epistaxis because the nasal blood vessels widen owing to the pressure of all the new circulating blood traveling throughout their bodies. The blood arteries in the nose are highly fragile and easily break (1). Severe nosebleed is a rare occurrence during pregnancy (3). Pregnant women who do not exhibit risk factors, such as the use of anticoagulant treatment or blood coagulation abnormalities, are comparatively less prone to have severe epistaxis (4). The pandemic during the 2019-2020 era, known as the coronavirus pandemic or COVID-19, was triggered by the emergence of severe acute respiratory syndrome coronavirus 2 (SARS-CoV-2). The initial identification of the virus occurred in Wuhan, China, during December in the year 2019 (5). On January 30, 2020, the World Health Organization (WHO) officially classified the outbreak as a public health emergency of international concern. Subsequently, on March 11, 2020, the WHO defined it as a pandemic (6).

## Materials and Methods

The sample for this study consisted of pregnant women who attended the Gynecologic and Obstetrics Department / Al-Yarmouk Teaching Hospital and Al-Karkh Maternity Hospital between May 2020 and April 2021. All were asked to complete a questionnaire about epistaxis and whether they had COVID-19 infection throughout this pregnancy. Only 3,362 pregnant women were included in the study and completed the questionnaire.

*Inclusion criteria*

Any pregnant women in the second and third trimesters who attended the Gynecologic and Obstetrics Department / Al-Yarmouk Teaching Hospital and Al-Karkh Maternity Hospital in the selected period.

*Exclusion criteria*

1. Women who are not currently pregnant.
2. First-trimester pregnant woman.
3. Epistaxis due to trauma or recent nasal surgery within two months of epistaxis.
4. Epistaxis is caused by blood diseases or drugs other than those taken for obstetrical or COVID-19 infection.

## Results

**Fig. 1:**
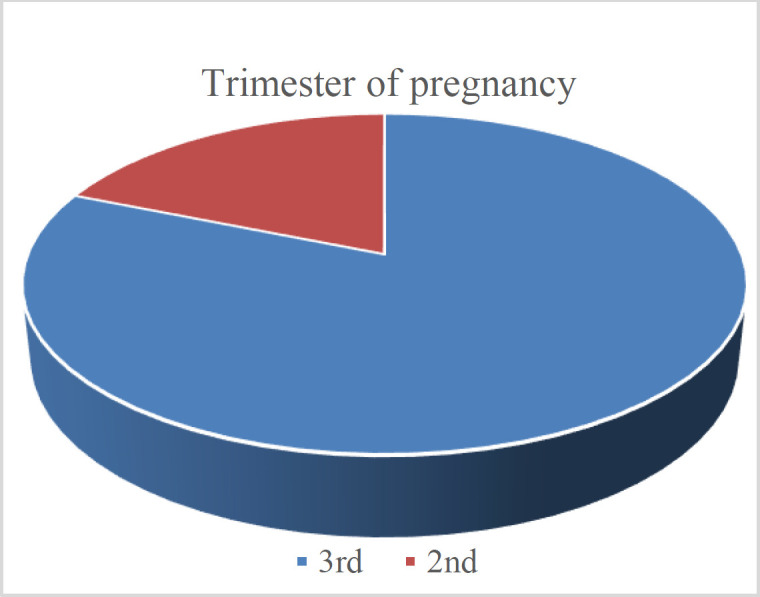
Trimesters of pregnancy between women involved in the study.

The questionnaire was submitted to every pregnant woman in the second and third trimesters who attended the Gynecologic and Obstetrics Department / Al-Yarmouk Teaching Hospital and Al-Karkh Maternity Hospital. The questionnaire was delayed for any emergency case or a patient in labor until her health condition stabilized. Three thousand three hundred sixty-two pregnant women were involved in this study between May 2020 and April 2021. The median sample age was 23.2 years. Two thousand seven hundred twenty-nine pregnant ladies are in the third trimester, and 633 are in the second trimester (Fig. 1).

Any pregnant women with multiple visits to the hospitals included in this study and the specified period of the study participated in more than one questionnaire; only the last questionnaire is counted.

*Statistical Analysis*

The questionnaires were collected and archived on a personal laptop. The data was first converted into SPSS v24, a statistical software for social sciences files, for analysis after being saved in Excel. The predetermined threshold for statistical significance, as shown by the P value, was established at 0.05 or less.

## Results

 After conducting statistical analyses, the result appeared as follows: 941 pregnant women (28%) had experienced an episode of epistaxis in the last pregnancy. 52% (1748 pregnant women) had a corona-positive history. 

**Fig. 2:**
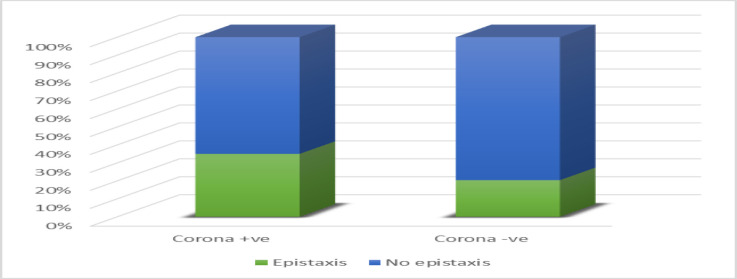
Epistaxis in pregnant women with positive and negative history of Corona.

 Pregnant women with a positive history of coronavirus infection have a higher incidence of epistaxis (612 pregnant women, 35%) than pregnant women with a corona-negative history of 20% (329 pregnant women), with a significant P value (0.039%) (Fig. 2).

**Fig. 3:**
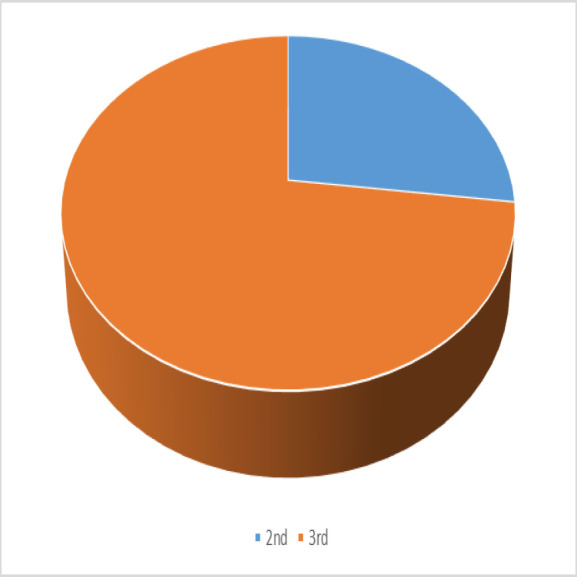
Distribution of epistaxis between pregnancy trimesters.

About two-thirds of pregnant women with nasal bleeding (687 pregnant women, 73%) develop attacks of epistaxis during the third trimester of pregnancy (Fig. 3).

**Fig. 4:**
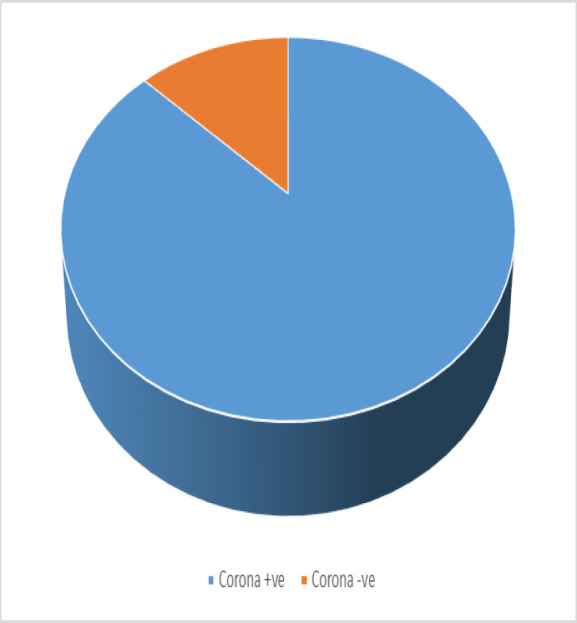
pregnant women with multiple epistaxis

Eighty-five pregnant ladies (9%) have more than one attack of nasal bleeding in the last pregnancy, and also, most of this group, 88% (74 pregnant ladies), have a coronavirus-positive history (Fig. 4).

**Fig. 5:**
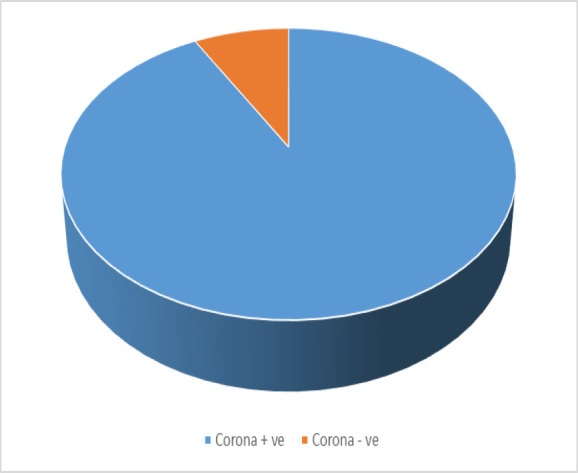
Coronavirus history among pregnant women with multiple epistaxis.

32% (301 pregnant ladies) have a history of epistaxis in a previous pregnancy. About two-thirds of them (198 pregnant women) mentioned that the epistaxis in this pregnancy appears to be more severe or more frequent than in the previous pregnancy. Also, most of the last group (182 pregnant women, 92%) have a corona-positive history (Fig.5). 92% mentioned that they had no epistaxis outside of pregnancy.

Only two patients gave a history of severe epistaxis that necessitated hospital admission, and one of them received blood. Moreover, both patients were infected with the Coronavirus.

## Discussion

Epistaxis is a frequently encountered issue in otolaryngology, particularly prevalent during pregnancy. Its management by healthcare personnel during the COVID-19 era may provide a potential risk, as it necessitates the proper utilization of personal protective equipment and adherence to recommendations (7). Our study showed an increase in nosebleeds among people with Coronavirus. It cannot be confident that epistaxis is a complication of infection with this virus, or it may be a symptom that helps diagnose it. Several things must be discussed to comprehend this relationship and its causes. 

Firstly, the use of Oxygen in some people infected with this virus may increase the likelihood of causing the dry lining of the nose, especially since a high percentage of infected people confirmed their use of Oxygen even in their homes as soon as they feel a kind of shortness of breath even without a lack of oxygen level in the blood and without medical advice. The utilization of Oxygen in managing COVID-19 pneumonia may have contributed to this elevated prevalence of epistaxis. Certain patients were administered Oxygen that was not humidified, either through a face mask or a low-flow nasal cannula. The administration of these treatments has been identified as an established risk factor for nasal dryness (8-10). 

Furthermore, extended oxygenation has been found to elevate the likelihood of crust development, compromising the integrity of the nasal mucosa and heightening the patient's susceptibility to nasal bleeding.

The study identified anticoagulant drugs as the second risk factor. These drugs may be used due to complications or risk factors for pregnancy or infection with the Coronavirus. Low-molecular-weight heparin (LMWH) was administered to specific individuals in our study, either for thromboprophylaxis or as a therapeutic intervention, based on their pregnancy risk factors or problems.  The consideration of this matter is vital due to the widespread utilization of LMWH in managing pregnant women with limited mobility and people afflicted with COVID-19 (11). Patients using such medications for reasons other than those mentioned above, for example, for blood clotting diseases, are excluded from the study.

Also, it was observed that some patients infected with the Coronavirus used one or more doses of such medicines, even without medical advice, sometimes, once an investigation showed a rise in the D-dimer analysis.

Two hundred sixty-nine patients in our study confirmed that they used subcutaneous injections of blood thinners due to high D-dimer as line management of COVID-19. Thirty-one patients received LMWH for obstetrical problems; all of these groups receiving blood thinner drugs developed epistaxis in our study. More observational studies are needed to support this idea because the sample size is too small to be conclusive.It is postulated that those who exhibit both risk factors are at an elevated risk for experiencing epistaxis. Another risk factor observed in this study related to the period of the Corona epidemic is the occurrence of nosebleeds after conducting a Corona test to take a nasal swab. Three cases were diagnosed, given such a history as a cause of epistaxis.

## Conclusions

The essential conclusion drawn from this study is the increase in the rate of nosebleeds in women during pregnancy. This point coincides with several studies on the same topic, including [Epistaxis of Pregnancy and Association with Postpartum Hemorrhage. Dugan-Kim et al.], [The way a nose could affect pregnancy: severe and recurrent epistaxis Laura Giambanco et al.] and [Torrential epistaxis in the third trimester: a management conundrum. Crunkhorn RE, Mitchell-Innes A, Muzaffar J.].

It was also observed that the rate of nosebleeds increased among pregnant women during the Corona pandemic period for several reasons, including:

1. Excessive use of oxygen causes dryness of the nose lining and increases the rate of nosebleeds. In addition, it is recommended to frequently prescribe moisturizing or lubricant substances to prevent atrophic rhinitis and the subsequent occurrence of epistaxis. Using humidified Oxygen is the best way.
2. The use of blood-thinning drugs and their direct effect on blood clotting in the nose.
3. Other reasons, such as the Corona swab from the nose, may cause a wound to the lining of the nose when performed by people with little experience and training.

Because the clinical assessment and treatment of patients with nasal bleeding commonly involves open nasal suction and may need a nasal plug, healthcare personnel who manage epistaxis patients are in danger of SARS-CoV-2 infection by the generated infected aerosols (12). Epistaxis prevention is still the best strategy for preventing or avoiding the abovementioned risk. It is noteworthy to mention that vaginal delivery has the potential to elevate the likelihood or intensity of nosebleeds, particularly in individuals with high-risk factors or a recent record of epistaxis. This is due to the exertion involved, the forceful pushing during the latter part of the second stage of labor, and the potential for Valsalva maneuvers to reinitiate epistaxis (13), an emergent or elective cesarean section could be the best choice.

## Strengths and Limitations

Because the short duration of patient data collection may affect some results, we recommend lengthening the period and expanding the sample size in future studies.
